# Enhanced secretion of thermostable phytases from *Myceliophthora thermophila* by *Komagataella phaffii*

**DOI:** 10.1186/s12934-026-02988-x

**Published:** 2026-04-22

**Authors:** Ronja Gratzer, Magdalena Merkaš, Andrej Nikolikj, Andreas Winkler, Martina Geier, Anton Glieder, Anita Emmerstorfer-Augustin

**Affiliations:** 1 Institute of Molecular Biotechnology, Graz University of Technology, NAWI Graz, Graz, Austria; 2 Institute of Biochemistry, Graz University of Technology, NAWI Graz, Graz, Austria; 3 bisy GmbH, Hofstaetten/Raab, Austria; 4 Austrian Centre of Industrial Biotechnology, acib GmbH, Graz, Austria; 5 BioTechMed-Graz, Graz, Austria

**Keywords:** *Komagataella phaffii*, Phytase, Phytate, Thermostability, Protein secretion, Signal sequences

## Abstract

Phytases catalyze the hydrolysis of phytic acid to release inorganic phosphate, thereby improving phosphorus bioavailability. Recombinant phytases hold great potential as additives in the feed, food, pharmaceutical, and chemical industries. However, their enzymatic stability and activity can be compromised during industrial processing, particularly during high-temperature treatments such as tableting. In this study, three putative phytase genes (designated *MtPhyA1*, *MtPhyA2*, and *MtPhyA3*) were identified from the thermotolerant fungus *Myceliophthora thermophila* through in silico sequence analysis. The genes were codon-optimized, cloned, and heterologously expressed in the methylotrophic yeast *Komagataella phaffii*. To enhance secretion efficiency, the native signal sequences were truncated and exchanged for the truncated *S. cerevisiae α*-mating factor signal peptide (Δ57–70). Among the three variants, the strain expressing *MtPhyA2* exhibited the highest production and enzymatic activity, with an optimal pH of 6.0 and temperature of 65 °C and specific activities reaching 147.7 ± 1.56 U mg^− 1^ at 37 °C, and 210.5 ± 4.23 U mg^− 1^ at 65 °C. To further enhance secretion, the pre-signal sequence of the *α*-mating factor signal peptide was replaced with alternative pre-sequences. The *K. phaffii* Ost1, *Kluyveromyces lactis* Ost1, and *K. phaffii* Gcw28 pre-sequences, when combined with the truncated *S. cerevisiae* α-factor pro-sequence (Δ57–70), improved phytase secretion by up to 60%. Upscaling in Eppendorf Minifors bioreactors further led to phytase production of 1.6 g L^− 1^, demonstrating a promising strategy for the efficient and economical production of thermostable phytases.

## Introduction

Phytate (myo-inositol hexakisphosphate) is the principal storage form of phosphorus in plant-derived feed ingredients such as cereals, legumes, and oilseeds. However, monogastric animals (e.g., pigs, poultry, and fish) lack sufficient endogenous phytase activity to efficiently hydrolyze phytate, resulting in poor phosphorus bioavailability. Consequently, inorganic phosphorus must be supplemented in feed, which increases production costs and contributes to environmental issues such as eutrophication due to the excretion of undigested phytate. Beyond its role in phosphorus storage, phytate also acts as an antinutritional factor by chelating essential minerals and binding proteins, thereby reducing their bioavailability [1, 2]. To overcome these limitations, exogenous phytases (myo-inositol hexakisphosphate phosphohydrolases) are widely employed in animal nutrition. These enzymes catalyze the sequential hydrolysis of phytate, releasing inorganic phosphate and improving nutrient absorption [3]. Consequently, the biotechnological production of phytases has emerged as an important industrial strategy to improve feed efficiency and minimize environmental phosphorus pollution. Microbial phytases are particularly important because of their high stability and activity under the acidic conditions of the monogastric digestive tract [4].

Research into microbial phytases began in the 1960s, when *Aspergillus niger* was identified as a potent phytase producer [5]. Since then, a wide range of phytase-producing microorganisms has been identified [4]. Commercial phytase products are typically categorized based on their origin (fungal or bacterial), the position of the initial dephosphorylation on the myo-inositol ring (3-phytases or 6-phytases), and their formulation, such as being coated or uncoated, to improve resistance to thermal inactivation during pelleting [6]. Phytases, particularly the histidine acid phytases that dominate commercial feed applications, typically possess a molecular mass between 35 and 50 kDa, exhibit optimal catalytic activity at pH values ranging from 4.5 to 6.0, and remain stable at temperatures between 45 and 70 °C [7]. Large-scale production is achieved either by cultivating naturally phytase-producing microorganisms or through recombinant expression in optimized host systems. Current commercial phytase formulations include mainly 3-phytases (EC 3.1.3.8), typically from fungal species like *A. niger* and *Penicillium funiculosum*, and 6-phytases (EC 3.1.3.26), derived from the bacteria *Escherichia coli*, *Citrobacter braakii*, *Buttiauxella* spp., and *Yersinia* spp., as well as the fungus *Peniophora lycii* [6, 8, 9].

To ensure a steady and scalable supply of phytases for commercial use, recombinant production systems have become essential. Various microbial hosts have been evaluated for their suitability in expressing phytases, with *Escherichia coli* periplasmic phytase AppA (AppA) serving as a prime example [10]. It has been produced in conventional bacterial systems like *E. coli* [6], but also in more innovative and sustainable platforms like the hydrogen-oxidizing bacterium *Cupriavidus necator*, which enable CO_2_-based bioproduction [11, 12]. Despite these advances, food-grade filamentous fungi, such as *Trichoderma reesei* and *Aspergillus* spp., as well as yeasts like *Komagataella phaffii*, remain the predominant hosts for large-scale recombinant phytase production, including that of AppA [13, 7]. These eukaryotic systems are favored for their efficient protein secretion capabilities, scalability, and compatibility with industrial fermentation processes. Importantly, filamentous fungi and yeasts can perform post-translational modifications, such as glycosylation and disulfide bond formation, which are essential for ensuring phytase stability, proper folding, and enzymatic activity [14, 15]. Moreover, the use of food-grade hosts is crucial to satisfy regulatory requirements and to streamline the approval process for applications in animal feed and food production.

To meet the demands of animal nutrition, phytases must offer more than high expression yields and scalability. The ideal enzyme should combine multiple functional and economic traits: high catalytic efficiency for phosphate release under the variable pH conditions of the gastrointestinal tract, robust industrial-scale production, and, critically, thermal stability to withstand feed processing. Heat resistance is particularly important during formulation steps such as pelletizing or tableting, where enzymes are exposed to temperatures up to 85 °C for up to one minute and must retain activity during storage. Although many phytases, such as those from *A. niger*, exhibit moderate heat tolerance with optimal activity at 52–55 °C and pH ~ 5.5 [16], enhancing thermostability remains a major research objective. To enhance phytase thermostability, several engineering strategies have been explored. For *E. coli* phytase AppA, methods such as error-prone PCR and site-directed mutagenesis have yielded thermostable variants [17]. Disulfide bond engineering, particularly when paired with the co-expression of protein disulfide isomerase in *K. phaffii*, has also proven effective by promoting proper protein folding and enhancing thermal stability [18].

In this study, we pursued a complementary strategy to phytase engineering by sourcing thermostable enzymes from the naturally thermophilic fungus *Myceliophthora thermophila* - formerly classified as *Sporotrichum thermophile* or *Thermothelomyces thermophilus* [19]. Previous studies have shown that *M. thermophila* exhibits moderate endogenous phytase activity [20, 21]. Moreover, a recombinant 3-phytase from this species (rSt-Phy, sp|O00107.1), when expressed in *E. coli*, displayed an activity profile well-suited to the digestive conditions of monogastric animals and was considered particularly attractive for industrial feed applications [22]. Building on these observations, we selected three phytase genes from the *M. thermophila* genome and assessed their secretory expression in *K. phaffii* using the MFα(Δ57–70) signal sequence. One candidate, *Mt*PhyA2, exhibited exceptionally high thermostability, retaining full enzymatic activity after exposure to temperatures above 90 °C.

To enhance secretion efficiency, we systematically evaluated alternative signal peptides, focusing on pre-regions from well-secreted fungal and yeast proteins. Pre-Ost1 sequences from *K. phaffii* and *Kluveromyces lactis*, as well as selected Gcw-derived pre-domains, markedly improved secretion when fused to the MFα(Δ57–70) pro-region. Notably, several pre-signal variants increased *Mt*PhyA2 secretion by up to twofold compared to the classical MFα(Δ57–70) signal.

Together, these findings demonstrate that *Mt*PhyA2 is a highly active and intrinsically thermostable phytase and that targeted optimization of pre-signal sequences represents an effective strategy to further enhance recombinant phytase production in *K. phaffii*. This work highlights the potential of combining naturally thermostable fungal enzymes with refined secretion signals to meet industrial demands for robust, high-performance feed phytases.

## Materials and methods

### Strains and growth conditions

Cloning was done in *E. coli* Top10F’ (Thermo Fisher Scientific). The *K. phaffii* BSYBG11 strain from bisy GmbH (Hofstätten an der Raab, Austria) was used as a wild-type strain for strain constructions. Cultivations were conducted in Luria Broth (LB) media for *E. coli* and yeast cultures were either grown in YPD medium (1% w/v yeast extract, 2% w/v peptone and 2% w/v glucose) or buffered minimal dextrose (BMD) medium (1.34% Yeast Nitrogen Base YNB, 4 × 10^− 5^% biotin, 200 mM potassium phosphate buffer pH 6.0 and 2% glucose). When required, Zeocin (Invitrogen) was added at 25 µg/mL for *E. coli* or 100 µg mL^− 1^ for *K. phaffii*. Protein production was induced by adding buffered minimal methanol (BMM) medium (1.34% YNB, 4 × 10^− 5^% biotin, 200 mM potassium phosphate buffer pH 6.0) with either 1% methanol (BMM2) or 5% methanol (BMM10). The first induction was performed with BMM2 (1% methanol), followed by subsequent inductions with BMM10 (5% methanol) every 12 h, resulting in a final methanol concentration of approximately 0.5% in the culture. Small-scale *K. phaffii* cultivations were performed in deep-well-plates (DWP) or 250 mL shake flasks using induction protocols described previously [23].

### Cloning and transformation of *K. phaffii*

Genome engineering of *K. phaffii* was performed using three strategies: (i) random integration, (ii) targeted integration at the *HIS4* locus, and (iii) replacement of the MFα(Δ57–70) signal sequence in already integrated constructs. For targeted integration into the *HIS4* locus, the pHIS4 vector was used [24]. Random genomic integration, including expression cassettes encoding *Candida antarctica* lipase B (CalB) and horseradish peroxidase (HRP), was achieved using the pPpT4-MFα(Δ57–70) vector [25]. For direct replacement of the MFα signal sequence upstream of expression cassettes, overlap extension PCR-generated repair cassettes were employed in combination with CRISPR/Cas9 [24].

The *M. thermophila* phytase genes were codon-optimized for *K. phaffii* and synthesized by Twist Bioscience. All DNA amplifications were carried out using Phusion™ High-Fidelity DNA Polymerase (Thermo Fisher Scientific) according to the manufacturer’s instructions. Plasmids were assembled by Gibson Assembly [26], fused to different signal sequences upstream of the phytase genes, and expressed under the control of the catalase promoter (*P*_*CAT*_) or alcohol oxidase 1 promoter (*P*_*AOX1*_). CalB and HRP were expressed under the control of *P*_*CAT*_. All phytase genes were expressed with a C-terminal hexahistidine (His₆) tag to facilitate purification and detection. Constructs were verified by sequencing and linearized with *Smi*I prior to transformation of *K. phaffii* BSYBG11 (bisy GmbH, Hofstätten an der Raab) by electroporation as described previously [27]. A list of plasmids and strains constructed and used in this study is provided in the Supplementary File.

### Recombinant gene expression

Recombinant protein production was performed in 250 mL baffled shake flasks following standard expression conditions at 28˚C, 250 rpm. The culture grew in 50 mL of BMD for 65 h. Methanol induction was then started by adding buffered minimal methanol medium (BMM2 or BMM10). During the 48 h induction phase, methanol was supplied every 12 h by pulse addition of BMM2 or BMM10, each containing defined methanol concentrations (1% or 5%, v/v). Volumes were calculated to maintain an approximate methanol concentration of 0.5% (v/v) in the culture until harvest at 112 h.

### SDS-PAGE and immunoblot analysis

Proteins from culture supernatants (10 µL) were prepared by adding 4 µL of 4 x NuPAGE™ sample buffer (Invitrogen) and 2 µL of NuPAGE™ reducing agent (Invitrogen).

Whole-cell extracts were obtained from 4 OD₆₀₀ units of cells, lysed in 300 µL of 1.85 M NaOH containing 7.5% β-mercaptoethanol on ice for 10 min, followed by precipitation with 300 µL of 50% trichloracetic acid for 1 h on ice. Pellets were washed and resuspended in 50 µL SDS loading buffer.

For SDS-PAGE, 10 µL of whole-cell lysates or 15 µL of supernatant samples were separated on NuPAGE™ Bis-Tris 4–12% gels (MES buffer) at 110 V for 50–60 min, alongside 5 µL of Spectra™ Multicolor Low Range Protein Ladder. Proteins were transferred to 0.45 μm nitrocellulose membranes at 10 V for 60 min. Membranes were blocked with 5% bovine serum albumin (BSA) in Tris-buffered saline with 0.1% Tween-20 (TBST) for 2 h, incubated overnight at 4 °C with HRP-conjugated anti-His antibody (1:1,500) in 2.5% BSA-TBST, and washed with TBST. Signal detection was performed using Clarity Max Western ECL and visualized using a G: Box imaging system.

### Bioreactor cultivation

Pre-cultures were prepared one day prior to bioreactor inoculation in 50 mL YPD medium. Exponentially growing *K. phaffii* cells were used to inoculate 500 mL of batch medium in 2 L bioreactors (Eppendorf SciVario twin system, DASGIP SR1000ODLS vessels) to an initial OD₆₀₀ of 1.5. The batch medium consisted of 40 g L⁻¹ glycerol, 0.64 g L⁻¹ KOH, 2.86 g L⁻¹ K₂SO₄, 0.22 g L⁻¹ NaCl, 2.32 g L⁻¹ MgSO₄·7 H₂O, 0.17 g L⁻¹ CaSO₄·2 H₂O, and PTM1 trace salts (4.35 mL L⁻¹). Batch cultivations were performed at 28 °C and pH 5.5, controlled using 25% (v/v) ammonium hydroxide. Dissolved oxygen (DO) was maintained at 30% air saturation by automatically adjusting agitation (600–1600 rpm) and aeration (30–80 standard liters per hour, sL h⁻¹). Antifoam (Antifoam 204, Sigma-Aldrich) was added automatically upon foam detection. PTM1 trace salts (6.0 g L⁻¹ CuSO₄·5 H₂O, 0.08 g L⁻¹ NaI, 3.0 g L⁻¹ MnSO₄·H₂O, 0.2 g L⁻¹ Na₂MoO₄·2 H₂O, 0.02 g L⁻¹ H₃BO₃, 0.5 g L⁻¹ CoCl₂, 20.0 g L⁻¹ ZnCl₂, 65.0 g L⁻¹ FeSO₄·7 H₂O, 0.2 g L⁻¹ biotin, and 5.0 mL L⁻¹ H₂SO₄) were included in both batch and feed media at a final concentration of 4.25 mL L⁻¹. The glycerol fed-batch phase was initiated upon depletion of glycerol, indicated by a sharp increase in DO. A 50% (w/v) glycerol feed supplemented with PTM1 trace salts was applied starting at 3 mL h⁻¹ and linearly increased to 10 mL h⁻¹ over 15 h (µ ≈ 0.08 h⁻¹), until approximately 100 mL of feed was consumed. DO was maintained at 30% throughout the fed-batch phase. Methanol induction was initiated following complete glycerol depletion, approximately 45 h after start of cultivation. Methanol adaptation was performed by pulsed addition to a final concentration of approximately 0.5% (v/v), followed by continuous feeding of 100% methanol supplemented with PTM1 trace salts at an initial rate of 1 mL h⁻¹. The methanol feed rate was subsequently adjusted based on DO levels to maintain methanol-limited conditions and prevent accumulation. Methanol feeding was maintained for approximately 47 h, until the end of cultivation. Cultures were monitored daily, and samples were collected for OD₆₀₀, dry cell weight (DCW), and phytase activity measurements. Cultivations were terminated on day 5 (92 h after start of cultivation), and cultures were harvested by centrifugation.

### Purification of His₆-tagged proteins

The secreted His-tagged *Mt*PhyA2 enzyme was purified by immobilized metal affinity chromatography (IMAC) using an ÄKTA pure system (Cytiva). The culture supernatant was first clarified by centrifugation and filtration (0.45 μm), and the 20 mL of the resulting sample was loaded onto a 5 mL HisTrap HP column (Cytiva) pre-equilibrated with binding buffer composed of 50 mM potassium phosphate (KPi), 500 mM NaCl, pH 7.4. Elution was performed using a linear gradient of imidazole (0–500 mM) in the same buffer system, with the elution buffer containing 50 mM KPi, 500 mM NaCl, and 500 mM imidazole, pH 7.4. Fractions containing the target protein were pooled and subjected to buffer exchange into 250 mM sodium acetate buffer (pH 5.5) using a 10 kDa molecular weight cut-off (MWCO) centrifugal ultrafiltration device (Amicon Ultra). The sample was concentrated to approximately 1.5 mL and diluted with fresh buffer, and this step was repeated three times at 4 °C to ensure complete buffer exchange (< 1 mM imidazole). The purity of the eluted protein was verified by SDS–PAGE.

### Determination of the protein concentration

Protein concentrations of purified enzyme samples were measured using the Bradford assay. For this, 20 µL of buffer-exchanged enzyme was mixed with 200 µL of Bradford reagent, incubated for 10 min at room temperature, and absorbance was read at 595 nm. Protein concentrations were calculated from a standard curve prepared with BSA. The same assay was also used to measure protein concentrations in clarified culture supernatants during bioreactor cultivation.

### Enzyme activity assays

#### Phosphatase activity

*Para*-nitrophenylphosphate assay: Acid phosphatase activity was assayed to measure phytase expression levels using an assay based on the substrate *para*-nitrophenylphosphate (*p*-NPP) (Sigma) at an initial concentration of 5 mM in the assay [28]. For the assay, 10 µL of culture supernatant was incubated with 190 µL of 5 mM *p*-NPP in 250 mM sodium acetate buffer (pH 5.5) for 1 h at 37 °C. The release of para-nitrophenol was quantified by measuring absorbance at 410 nm using a NanoDrop spectrophotometer. All reactions were performed in triplicate in 96-well plates. One unit (U) of enzyme activity was defined as the amount of enzyme required to release 1 µmol of para-nitrophenol per minute under the specified assay conditions. Activity was expressed as units per milliliter of enzyme solution (U·mL⁻¹) and normalized to cell density (U·mL⁻¹·OD_600_⁻¹). Activity values were calculated according to the Beer–Lambert law using the extinction coefficient at 410 nm (ε₄₁₀ = 18,500 M⁻¹·cm⁻¹), taking into account reaction volume, sample volume, optical path length, incubation time, and dilution factors. Normalization to OD_600_ compensates for minor differences in cell growth between samples. The *p*-NPP assay served as a rapid screening and comparative method to evaluate expression and secretion efficiency of the same phytase (*Mt*PhyA2) expressed with different secretion signal variants. It should be noted that the *p*-NPP assay provides relative activity measurements and does not reflect absolute phytase activity.

#### Phytase activity

Phytate assay: Crude protein samples were initially subjected to buffer exchange into phytase assay buffer (0.25 M sodium acetate, pH 5.5) using a Superfine HiTrap Desalting Column (GE Healthcare), operated at a flow rate of 5 mL min^− 1^. Phytase activity was determined by quantifying the release of inorganic phosphate from sodium phytate, following a modified protocol based on Heinonen and Lahti [29]. In brief, 40 µL of appropriately diluted enzyme solution was mixed with 80 µL of 12 mM sodium phytate (substrate), and the reaction was incubated at 35 °C, 37–65 °C (depending on the experiment) for 10 min (SimpliAmp Thermal Cycler, Fisher Scientific). The reaction was terminated by adding 20 µL of 1 M HCl. Color development was achieved by adding a phosphomolybdate detection solution containing freshly prepared 10% acorbic acid as the reducing agent, and the mixture was incubated at room temperature for 10 min. The amount of liberated phosphate was quantified by measuring absorbance at 850 nm. The concentration of released inorganic phosphate was determined using a standard calibration curve prepared with potassium phosphate (K₂HPO₄) in the same assay buffer. One unit (U) of phytase activity was defined as the amount of enzyme that liberates 1 µmol of inorganic phosphate per minute under the specified assay conditions. All reactions were conducted in triplicate using 96-well plates.

#### Peroxidase activity

2,2′-Azinobis-(-3 ethylbenzothiazoline-6-sulfononic acid) diammonium salt (ABTS) assay: Enzyme activity was determined in 350 µL reaction mixtures containing 297.5 µL of 50 mM citrate–phosphate buffer (pH 4.5), 25 µL of ABTS (from a freshly prepared 20 mM stock), 2.5 µL of H₂O₂ (50 mM stock prepared in the same buffer), and 25 µL of enzyme sample (culture supernatant). Reactions were mixed gently and incubated at 35 °C. Oxidation of ABTS was monitored by measuring the increase in absorbance at 420 nm for 15 min at room temperature using a spectrophotometer (BioTek SynergyMX microplate reader from Agilent Technologies, USA). One unit of peroxidase (HRP) activity was defined as the amount of enzyme catalyzing the oxidation of 1 µmol of ABTS per minute under the assay conditions (ε_ABTS = 36.8 mM⁻¹ cm⁻¹). Data are reported as the mean ± standard deviation of at least three independent measurements.

#### Lipase activity

*Candida antarctica* lipase B (CalB) assay: CalB activity in the culture supernatant was determined by monitoring the hydrolysis of p-nitrophenyl butyrate (p-NPB) to p-nitrophenol (p-NP) at 405 nm (A405), similar to the assay described by Krainer et al. 2012 [30]. Briefly, 20 µL of culture supernatant was mixed with 180 µL of fresh assay solution containing 4 mM p-NPB in 300 mM Tris–HCl, pH 7.4, 1% acetone. The absorbance increase at 405 nm was monitored at room temperature for 15 min (BioTek SynergyMX microplate reader from Agilent Technologies, USA). One unit of lipase activity was defined as the amount of enzyme releasing 1 µmol p-nitrophenol per minute under assay conditions (εPNP = 0.0148 µM − 1.cm − 1). All lipase activity assays were performed in triplicates on two independent cultures.

All assays included appropriate blanks to account for background absorbance. For screening of cell supernatants, the blank was the supernatant from the wild-type BSYBG11 strain, which does not produce phytase, and was processed under the same conditions as the samples to serve as a negative control. For purified protein assays, the corresponding buffer was used as the blank.

### Determination of pH optimum, temperature optimum, and stability

pH optimum: The pH dependence of phytase activity was determined using the buffer-exchanged purified enzyme and following buffer system: 0.1 M citrate buffer for pH 3.0, 250 mM sodium acetate buffer for pH 4.0–6.0, and 250 mM Tris-HCl buffer for pH 7.0–8.0. Enzymes were incubated with 12 mM sodium phytate in the respective buffers at 65 °C for 10 min. Activity was measured as described and relative activity was calculated by normalizing all values to the maximum observed activity, which was defined as 100%.

Temperature optimum: The buffer-exchanged purified enzyme samples were incubated with 12 mM sodium phytate in 250 mM acetate buffer over 25–75 °C for 10 min. Activity was measured as described and relative activity was calculated by normalizing all values to the maximum observed activity, which was defined as 100%.

Thermal stability (heat shock): The buffer-exchanged purified enzyme samples were subjected to 75, 85, and 95 °C for 2 min, cooled to room temperature, and residual activity was assayed at 65 °C and 37 °C in 250 mM acetate buffer (pH 5.5). Activity was expressed as residual activity (%), normalized to the activity of the untreated control sample, which was defined as 100%.

### Specific activity calculation

The specific phytase activity was calculated by dividing the enzyme activity (U·mL^− 1^), determined using the phytate assay under standard conditions (250 mM sodium acetate buffer, pH 5.5, 37 °C and 65 °C, 10 min) and a phosphate standard calibration curve generated with KH₂PO₄, by the protein concentration of the purified enzyme (mg·mL^− 1^), determined using the Bradford assay. This yields activity in U·mg^− 1^, representing the enzymatic activity per milligram of total protein.

### Intact mass analysis

The mass spectrometry analysis was using Impact II UHR-TOF MS. Aliquots of 3 µL protein at 10 µM were desalted on a Shim-pack Scepter C4-300 (G) column (3 µM) (Shimadzu, Kyoto, Japan) by washing with a solution of 1% acetonitrile and 0.1% formic acid. Subsequently, a gradient of acetonitrile (1 to 95%) eluted the proteins into an Impact II ESI-Q-TOF (Bruker, MA, USA) mass spectrometer. Eluting protein signatures were integrated and deconvoluted using the maximum entropy function of DataAnalysis (Bruker, MA, USA).

## Results

### Phytases from *M. thermophila* are efficiently secreted by *K. phaffii*

**Fig. 1:**
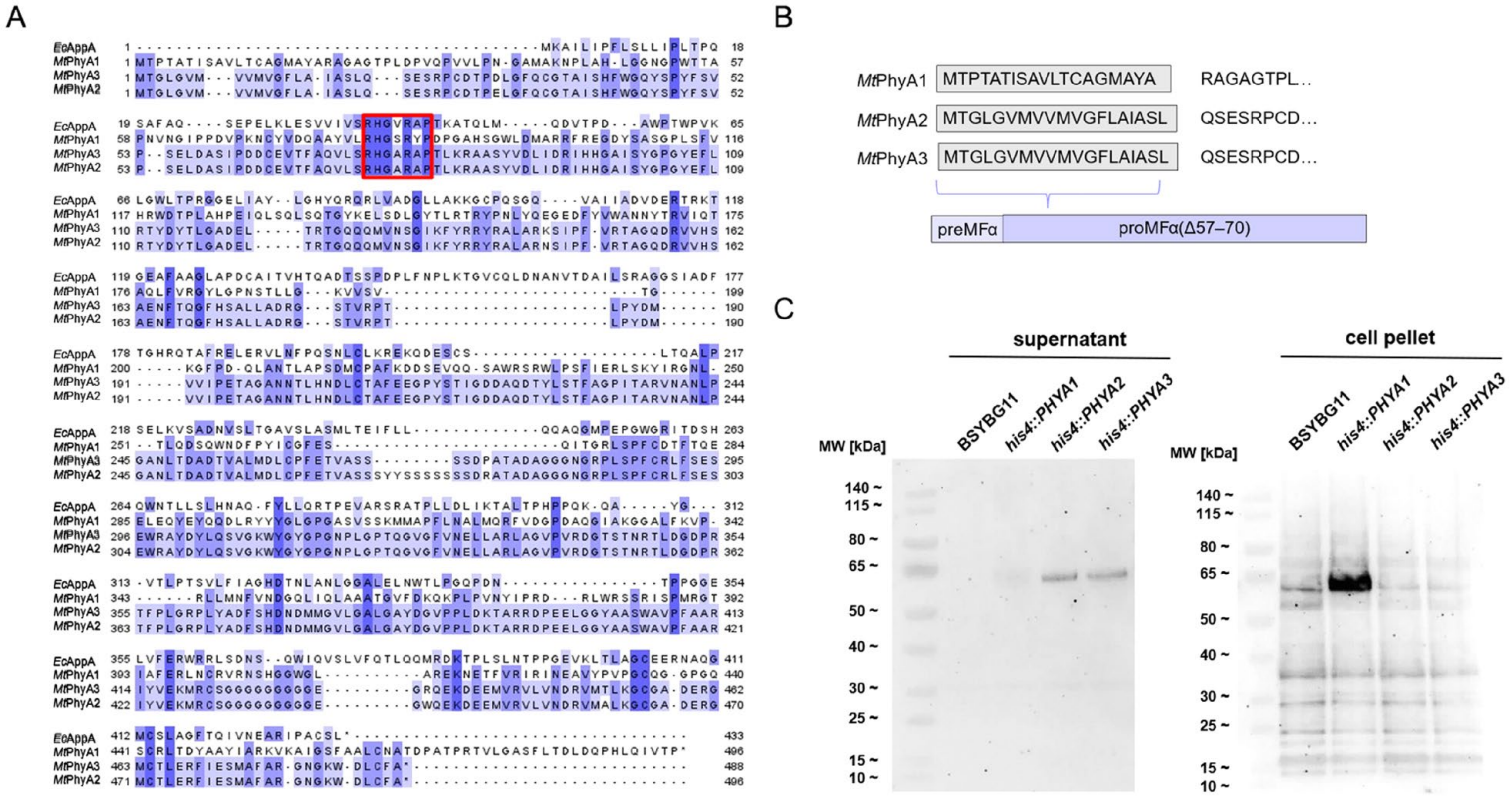
Secretion of phytases from *M. thermophila* in *K. phaffii*. **A** Multiple sequence alignment of AppA, *Mt*PhyA1, *Mt*PhyA2, and *Mt*PhyA3, performed using Clustal Omega [33] and visualized with Jalview [34]. Blue shading indicates the degree of amino acid conservation, with darker shading representing higher sequence conservation. The red box represents the conserved fingerprint motif (RHGxRxP) commonly found in the active site of histidine acid phytases [35]. **B** Schematic representation of signal sequence truncation and replacement with the MFα(Δ57–70) prepro signal sequence for all three phytase proteins. **C** Immunoblot analysis of phytase production. Strains BSYBG11 Δ*his4*::*P*_*CAT*_-*MtPHYA1*-His_6_ (yRG001), BSYBG11 Δ*his4*::*P*_*CAT*_-*MtPHYA2*-His_6_ (yRG003), and BSYBG11 Δ*his4*::*P*_*CAT*_-*MtPHYA3*-His_6_ (yRG005) were cultivated in BMD medium and induced with BMM2 as described in Materials and Methods. Proteins from six samples, including both supernatant and cell pellet fractions, were precipitated with TCA at 4 °C overnight. Phytases were detected using an anti-HIS antibody. Molecular weight markers (PageRuler™ Prestained Protein Ladder, Thermo Fisher Scientific) are indicated on the left. The expected molecular weight of the *Mt*PhyA proteins is ~ 55 kDa

Three phytase proteins were identified in the thermotolerant fungus *M. thermophila* based on sequence identity to *E. coli* AppA and in silico analysis: *Mt*PhyA1 (NCBI Reference Sequence: XP_003665855.1), *Mt*PhyA2 (NCBI Reference Sequence: XP_069297035.1), and *Mt*PhyA3 (UniProt ID O00107, also known as rSt-Phy [22]), (Fig. 1A). *Mt*PhyA2 and *Mt*PhyA3 are highly similar (98% squence identity), differing by only a few amino acids, whereas *Mt*PhyA1 shows lower overall sequence identity of 25%. ative N-terminal signal sequences, common in fungal phytases, were identified using SignalP 6.0 [31] and replaced with the *S. cerevisiae* MFα(Δ57–70) signal peptide [32] (Fig. 1B). All expression constructs were integrated into the *HIS4* locus of *K. phaffii*. Expression under the methanol-inducible *CAT1* promoter revealed that *Mt*PhyA2 and *Mt*PhyA3 were efficiently secreted into the culture supernatant, as indicated by immunoreactive bands at ~ 65 kDa (Fig. 1C). In contrast, *Mt*PhyA1 accumulated predominantly intracellularly, suggesting impaired secretion under these conditions. The higher apparent molecular weights compared to the ~ 55 kDa reported for *Mt*PhyA3 (rSt-Phy) produced in *E. coli* suggest that the enzymes are glycosylated in *K. phaffii*.

### Activity assessment of phytases from *M. thermophila*

**Fig. 2:**
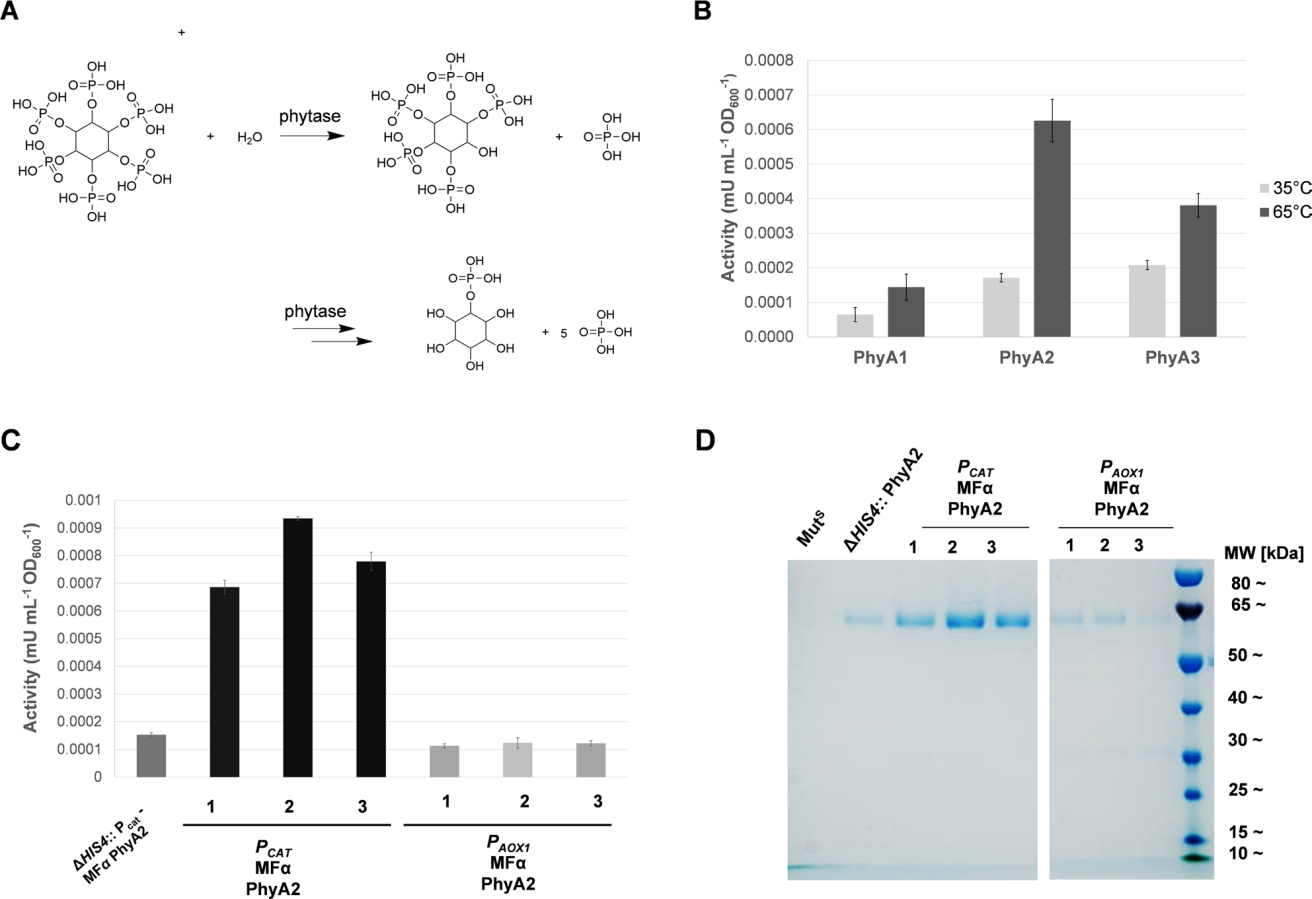
Phytases secreted by *K. phaffii* are active. **A** Schematic of the phytase-mediated hydrolysis of phytate (myo-inositol hexakisphosphate), releasing inorganic phosphate. **B** Strains BSYBG11 *Δhis4:: P*_*CAT*_*-MtPHYA1*-His6 (yRG001), BSYBG11 *Δhis4::P*_*CAT*_*-MtPHYA2-*His6 (yRG003), and BSYBG11 *Δhis4::P*_*CAT*_*-MtPHYA3*-His6 (yRG005) were cultivated and induced, and buffer-exchanged culture supernatants were used to determine activity using sodium phytate as substrate. **C** Strains BSYBG11 Δ*his4*::*P*_*CAT1*_-*MtPHYA2*-His_6_ (yRG003), BSYBG11 pPpT4-*P*_*CAT1*_-MFα(Δ57–70)-*MtPHYA2*-His_6_ (3 different clones: yRG0011, yRG0012, and yRG0013), and BSYBG11 pPpT4*-P*_*AOX1*_-MFα(Δ57–70)-*MtPHYA2*-His_6_ (3 different clones: yRG014, yRG015, and yRG016) were tested for phytate-dependent activity. **D** The same strains as in **C** were analyzed by SDS–PAGE and Coomassie staining. MW marker: PageRuler™ Prestained Protein Ladder, 10–140 kDa (Thermo Fisher Scientific, Cat. No. 26616). All cultivations and measurements were performed in biological triplicates; mean values and standard errors are shown

Activity assays with the natural substrate phytate (Fig. 2A), performed after buffer exchange to remove residual phosphate, confirmed that all three enzymes exhibited measurable phytate-dependent activity at 35 °C (Fig. 2B). Notably, all phytases showed increased phytate-dependent activity at 65 °C, with *Mt*PhyA2 exhibiting the best performance, identifying it as the most promising candidate for further optimization.

To enhance production, *Mt*PhyA2 was integrated randomly into the genome, and expression landscaping was performed to identify high-producing clones [36] (Supplementary Figure S1). Shake flask cultivations of the three best clones revealed a 5–6-fold increase in phytate-dependent activity and secretion levels compared with the *HIS4*-locus control (Fig. 2C, D). Replacement of the *CAT* promoter with the usually stronger, methanol-inducible *AOX1* promoter did not further improve secretion or relative activity, as confirmed by subsequent expression landscaping (Supplementary Figure S2) and shake flask analysis (Fig. 2C, D).

### *Mt*PhyA2 exhibits exceptional thermal and broad pH stability

**Fig. 3:**
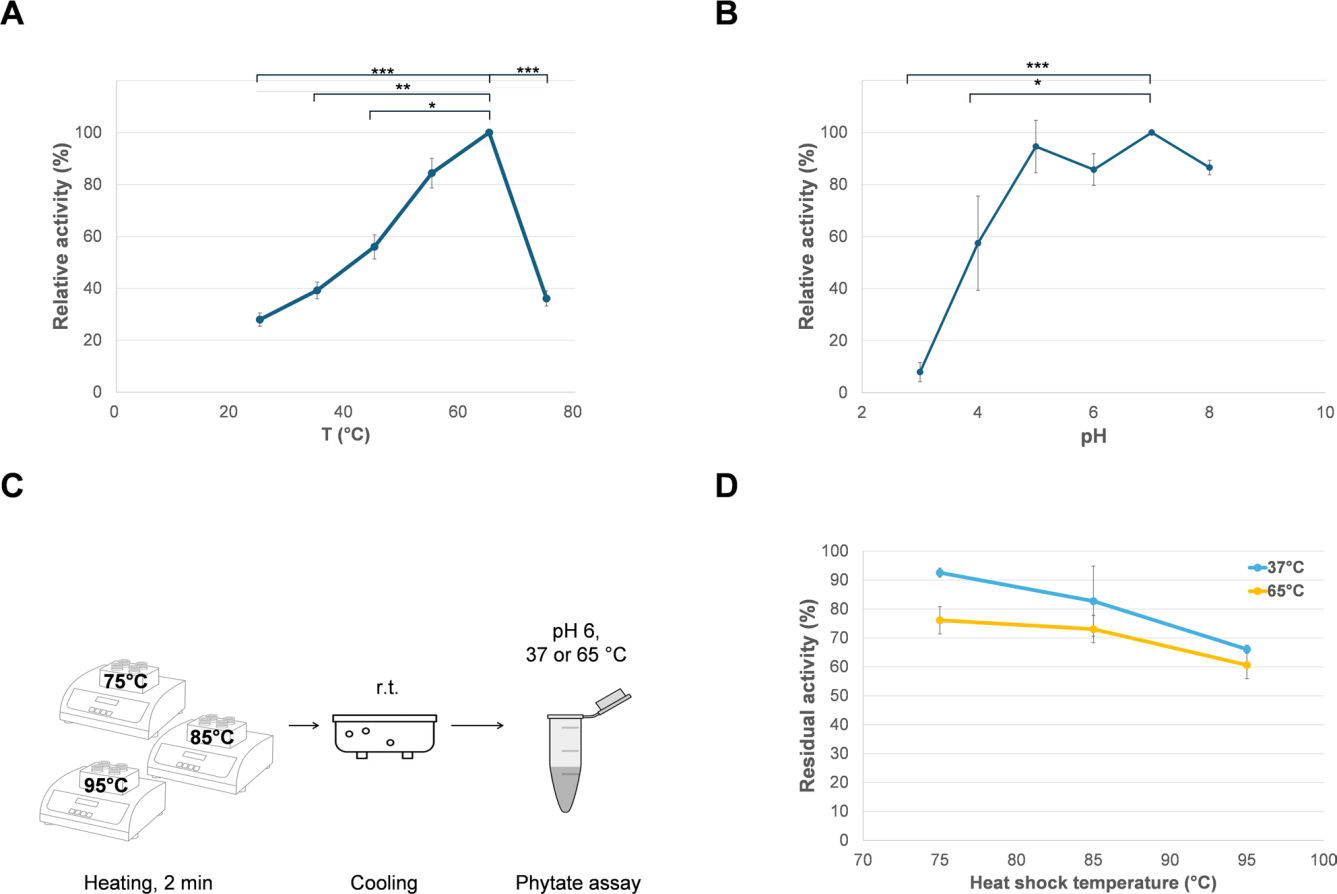
*Mt*PhyA2 is active at high temperatures and across a broad pH range.** A** Temperature profile of purified *Mt*PhyA2. Phytate-dependent activity was assayed using sodium phytate as substrate at temperatures ranging from 25 °C to 75 °C.** B** pH stability of *Mt*PhyA2. Enzyme activity was measured at pH values from 3 to 8 at 35 °C.** C** Schematic representation of the heat-shock assay simulating tablet processing conditions. Purified *Mt*PhyA2 was pre-incubated at various temperatures for defined time intervals to mimic tablet processing (> 80 °C for 1–2 min), followed by activity measurements at 37 °C (physiological temperature) and 65 °C (optimal temperature).** D** Residual activity of purified *Mt*PhyA2 after pre-incubation under the simulated tablet processing conditions described under C. All cultivations and measurements were performed in biological triplicates; mean values and standard errors are shown. Statistical significance was assessed using Student’s t-test. P-values are indicated as follows: *p* < 0.05 (*), *p* < 0.001 (**), and *p* < 0.0001 (***)

Ni-NTA purified *Mt*PhyA2 displayed phytate-dependent activity at 65 °C, while activity gradually declined at temperatures 10 °C lower or higher (25–75 °C) (Fig. 3A). Although activity at 65 °C was slightly higher than at 55 °C, this difference was not statistically significant (*p* = 0.07, t-test). pH profiling showed that *MtP*hyA2 maintained high catalytic activity between pH 5 and 8, while activity was reduced by ~ 50% at pH 4 and decreased further at pH 3 (Fig. 3B). To simulate the elevated temperatures encountered during tablet processing, which typically exceed 80 °C for 1–2 min [37], purified *Mt*PhyA2 was pre-incubated at different temperatures for defined time intervals, followed by activity measurements at 37 °C, representing physiological conditions in the mammalian stomach, and at 65 °C, corresponding to the enzyme’s optimal temperature (Fig. 3C). Remarkably, pre-incubation at temperatures up to 95 °C resulted in substantial retention (approximately 60%) of phytate-dependent activity, demonstrating the high thermostability of *Mt*PhyA2.

### Secretion of *Mt*PhyA2 can be improved by exchanging the MFα pre-signal sequence

**Fig. 4:**
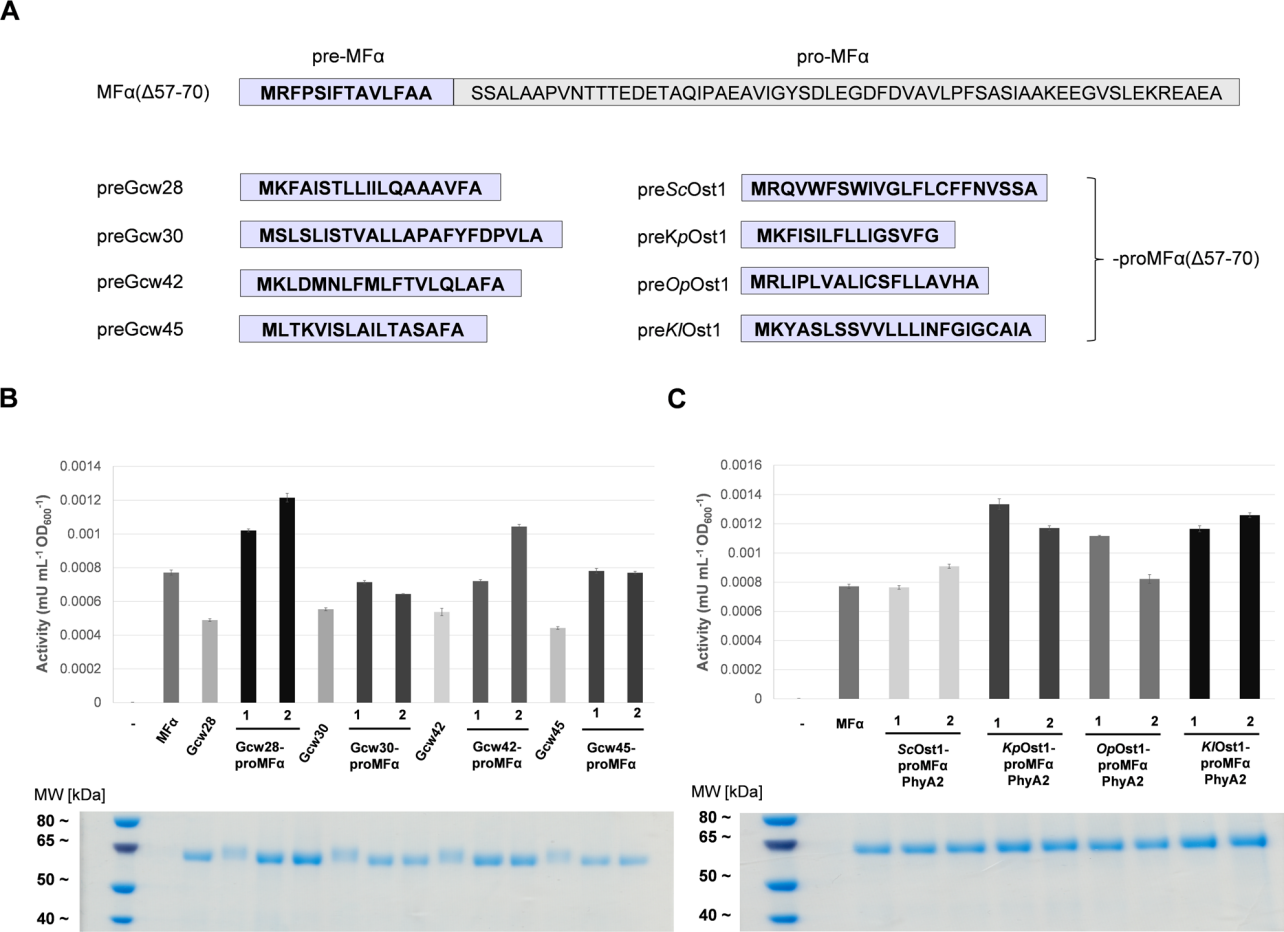
Improving secretion of *Mt*PhyA2 by combining different pre-signal sequences with the MFα(Δ57–70) pro-signal sequence.** A** Amino acid sequences of the signal peptides, including preproMFα(Δ57–70), preGcw28, preGcw30, preGcw42, preGcw45, pre*Sc*Ost1, pre*Kp*Ost1, pre*Op*Ost1 and pre*Kl*Ost1 signal sequence. Whenever indicated in the corresponding construct, the respective pre-signal sequence was fused upstream of the proMFα(Δ57–70) region to generate a chimeric pre–pro signal peptide; in other cases, only the pre-signal sequence was fused directly to *Mt*PhyA2.** B** The following Gcw signal sequences and strains were analyzed for secretion capacity on *p*NPP as substrate and by SDS–PAGE/Coomassie staining: BSYBG11, MFα–*Mt*PhyA2 (yRG011), preGcw28-*Mt*PhyA2 (yRG023), preGcw28–proMFα(Δ57–70)–*Mt*PhyA2 (two clones: yAN005, yAN006), preGcw30-*Mt*PhyA2 (yRG024), preGcw30–proMFα(Δ57–70)–*Mt*PhyA2 (two clones: yAN007, yAN008), preGcw42-*Mt*PhyA2 (yRG026), preGcw42–proMFα(Δ57–70)–*Mt*PhyA2 (two clones: yAN009, yAN010), preGcw45-*Mt*PhyA2 (yRG027), preGcw45–proMFα(Δ57–70)–*M*tPhyA2 (two clones: yAN011, yAN012).** C** The following Ost1 signal sequences and strains were analyzed for secretion capacity on *p*-NPP as substrate and by SDS–PAGE/Coomassie staining: BSYBG11, pre*Sc*Ost1–proMFα(Δ57–70)–*Mt*PhyA2 (two clones: yRG017, yRG018), pre*Kp*Ost1–proMFα(Δ57–70)–*Mt*PhyA2 (two clones: yRG031, yRG032), pre*Op*Ost1–proMFα(Δ57–70)–*Mt*PhyA2 (two clones: yAN001, yAN002), pre*Kl*Ost1–proMFα(Δ57–70)–*Mt*PhyA2 (two clones: yAN003, yAN004). All measurements were performed in biological triplicates; mean values and standard errors are shown. MW marker: PageRuler™ Prestained Protein Ladder, 10–140 kDa (Thermo Fisher Scientific, Cat. No. 26616) is indicated

Inspired by Zhang et al. [38], who analyzed C-terminal domains of GPI-anchored Gcw proteins for cell surface display of CalB in *K. phaffii*, we hypothesized that these proteins, efficiently localized to the cell surface, may harbor potent N-terminal secretion signals. Full-length sequences of selected Gcw proteins were analyzed, and native signal peptides were predicted using SignalP 6.0 [31] (Fig. 4A). To minimize clonal variation, CRISPR/Cas9-mediated genome editing with homology-directed repair was used to replace the MFα signal peptide in the best-performing strain yRG011. Secretion efficiency varied among Gcw pre-signals: Gcw19, Gcw21, Gcw22, Gcw51, and Gcw61 performed poorly, while others enabled ~ 30–50% of the secretion observed with MFα(Δ57–70) (Supplementary Figure S3). Mass shifts suggested incomplete or incorrect processing of all Gcw pre-peptides.

To improve processing, the four best-performing Gcw pre-signal coding sequences (*GCW28*, *GCW30*, *GCW42*, *GCW45*) were fused upstream to the MFα(Δ57–70) pro-sequence in pPpT4-*MtP*hyA2 vectors (Fig. 4A), and randomly integrated into the *K. phaffii* genome. Expression profiling (Supplementary Figure S4, S5, S6 and S7) identified the two highest-producing strains for shake flask cultivation. Similarly, Ost1 pre-signals from different hosts - including *S. cerevisiae* [39], *O. polymorpha*, and *K. lactis* - were fused upstream to the MFα pro-sequence (Fig. 4A). Following random integration and expression profiling (Supplementary Figure S8, S9 and S10), the two top-performing strains were selected for cultivation.

Overall, combining Gcw28 or *Kp*Ost1 pre-signals with the MFα(Δ57–70) pro-sequence substantially increased secretion compared with the pre-signals alone, and processing appeared correct, as indicated by the expected protein sizes (Fig. 4C). Among all tested combinations, the Gcw28–MFα fusion showed the highest improvement, increasing phytase production by approximately 60%.

**Fig. 5:**
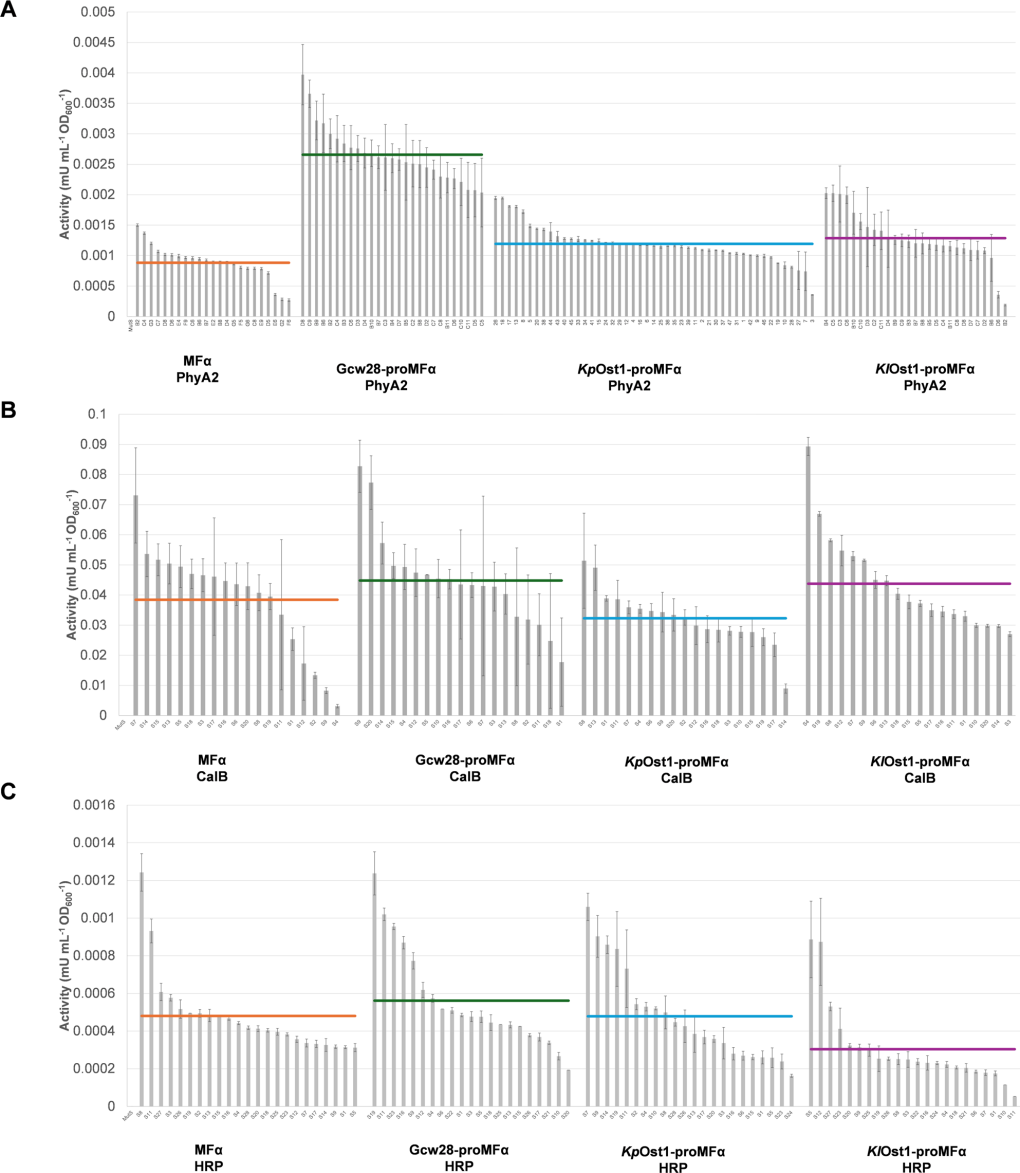
Comparison of secretion efficiency of different signal sequence combinations for three recombinant proteins. Expression profiling was performed for constructs containing the signal peptides MFα(Δ57–70), preGcw28–proMFα(Δ57–70), KlOst1–proMFα(Δ57–70), and KpOst1–proMFα(Δ57–70) directing secretion of (**A**) *Mt*PhyA2, (**B**) CalB, and (**C**) HRP in *K. phaffii* strain BSYBG11. All measurements were performed in biological triplicates; mean values and standard errors are shown. Colored horizontal bars indicate the overall mean activity across all clones measured for each variant in the expression landscape

To evaluate whether the engineered pre–pro signal sequence combinations also enhance secretion of other recombinant proteins, the respective signal peptides were fused upstream of the reporter enzymes CalB and HRP and compared with the standard MFα(Δ57–70) signal peptide. To assess the effects of the different signal sequences on secretion efficiency, expression landscapes were generated from multiple independent clones after random genomic integration and expression profiling (Fig. 5). For comparison, the corresponding expression landscapes for the same signal sequence combinations directing secretion of PhyA2 are also shown (Fig. 5A). Overall, the tested signal peptide combinations (preGcw28–proMFα(Δ57–70), *Kp*Ost1–proMFα(Δ57–70), and *Kl*Ost1–proMFα(Δ57–70)) barely improved secretion relative to MFα(Δ57–70). All constructs exhibited comparable performance for secretion of CalB (Fig. 5B) and HRP (Fig. 5C), indicating that these engineered signal peptides are broadly functional, but that their beneficial effect is most pronounced for *Mt*PhyA2.

### Upscaling of phytase production and comprehensive mass analysis

**Fig. 6:**
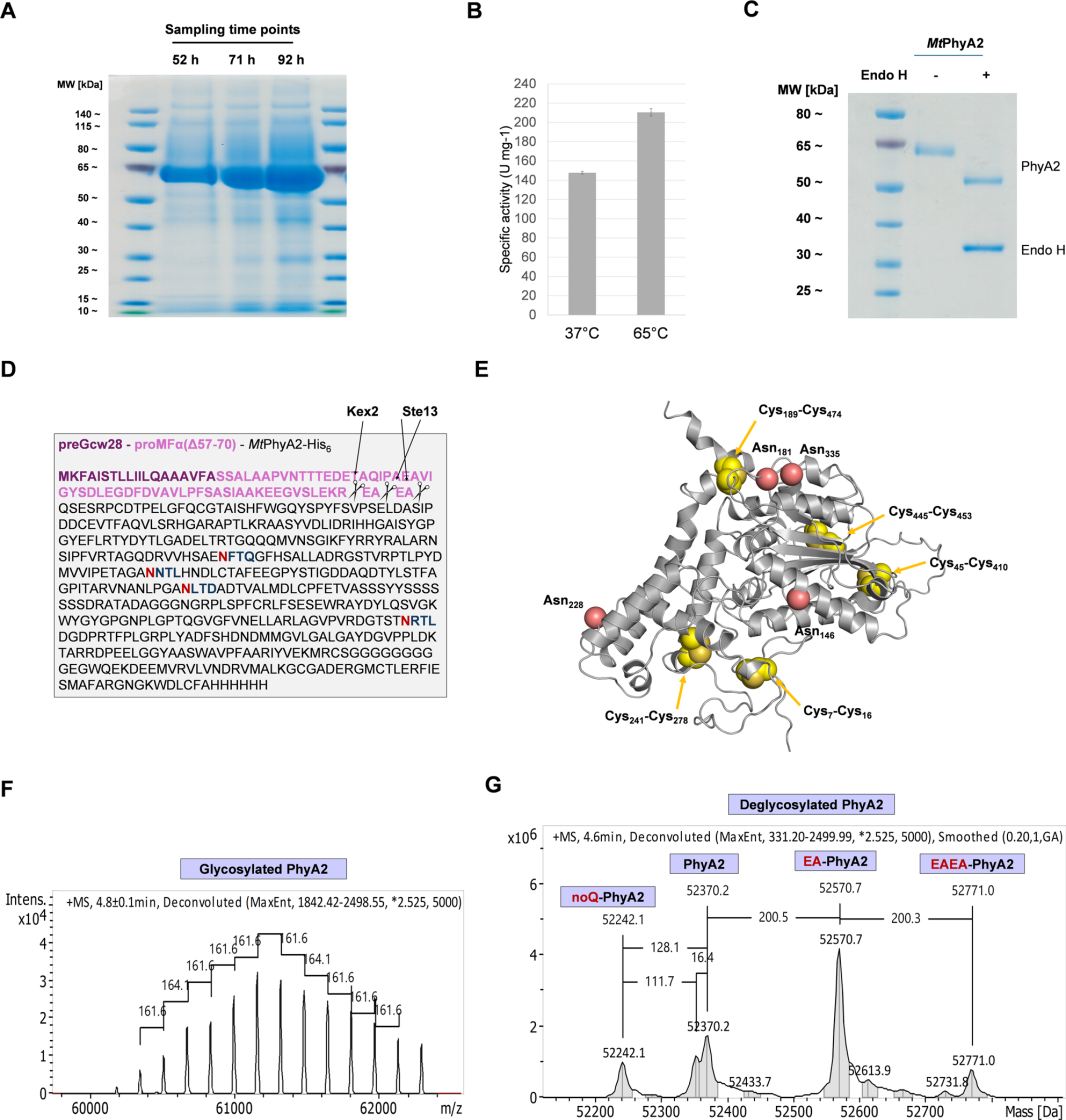
Characterization of *Mt*PhyA2 after bioreactor cultivation and purification. **A** Strain BSYBG11 pre*GCW28*–proMFα(Δ57–70)–*Mt*P*HYA2* (yAN006) was cultivated in a bioreactor, and supernatant samples taken at 52, 71, and 92 h of total culture time were directly analyzed by SDS–PAGE followed by Coomassie staining. MW marker: PageRuler™ Prestained Protein Ladder, 10–140 kDa (Thermo Fisher Scientific, Cat. No. 26616). **B** Specific activity (U mg⁻¹) of *Mt*PhyA2 purified from culture supernatants harvested after 92 h of bioreactor cultivation. **C** SDS–PAGE analysis and Coomassie staining of purified *Mt*PhyA2 treated with or without Endo H. MW marker: PageRuler™ Prestained Protein Ladder, 10–140 kDa (Thermo Fisher Scientific, Cat. No. 26616). **D** Primary amino acid sequence of pre-Gcw28 (purple), pro-MFα(Δ57–70) (violet), and *Mt*PhyA2–His₆. Predicted Kex2 and Ste13 cleavage sites are indicated by scissors. Predicted N-glycosylation recognition motifs are highlighted in bold and asparagine residues predicted to be N-glycosylated are shown in red. **E** AlphaFold3 structural model of *Mt*PhyA2 displaying the five predicted disulfide bonds and the four asparagine residues predicted to be glycosylated. **F** Total mass spectrum of *Mt*PhyA2 before Endo H treatment. **G** Total mass spectrum of *Mt*PhyA2 after Endo H treatment

The best-performing strain expressing pre*GCW28*-proMFα(Δ57–70)-*MtPHYA2* was further evaluated in controlled bioreactor cultivations. After 92 h of total culture time, the dry cell weight reached 126.9 g L⁻¹ (Supplementary Figure S11), with a corresponding phytase titer of approximately 1.6 g L⁻¹ as determined from enzyme concentrations measured by the Bradford assay at the harvesting (Fig. 6A). Purified *Mt*PhyA2 was subsequently assayed, revealing intrinsic specific activities of 147.7 ± 1.56 U mg⁻¹ at 37 °C and 210 ± 4.23 U mg⁻¹ at 65 °C (Fig. 6B).

To investigate the extent of glycosylation, samples were deglycosylated and analyzed by SDS-PAGE, revealing a clear mass shift compared with untreated protein (Fig. 6C). Potential N-linked glycosylation sites were predicted using NetNGlyc 1.0 [40], identifying four asparagine residues within the conserved N-X-S/T motif (X ≠ Pro): N146, N181, N228, and N335 (Fig. 6D). Among these, N146 and N228 showed the highest prediction scores and full jury agreement, indicating the greatest likelihood of glycosylation. Based on the observed molecular mass shift, it is most likely that only one, or at most two, sites are occupied, presumably one of the two highest-scoring residues (N146 or N228). Structural modeling with AlphaFold further predicted the presence of five disulfide bonds (Fig. 6E).

Mass spectrometry (MS) analysis of the purified enzyme confirmed extensive N-glycosylation (Fig. 6F). The intact mass spectrum showed *Mt*PhyA2 as a heterogeneous mixture of glycoforms, with microheterogeneity in mannose content evident from peaks spaced by ~ 161 Da. Following Endo H treatment, the spectrum revealed several distinct species corresponding to alternatively processed N-termini. The theoretical mass of fully processed *Mt*PhyA2 is 52,367 Da (including the − 10 Da contribution from disulfide bond formation). The closest measured peak was detected at 52,370 Da (+ 3 Da), potentially indicating that not all 5 predicted disulfide bonds are formed. A minor peak 16.4 Da lower than the 52,370 Da species was also detected, which may indicate partial N-terminal pyroglutamate formation [41]. A second species at 52,242 Da corresponded to removal of the N-terminal glutamine residue. Additional peaks approximately + 200 Da above the expected mass were assigned to proteins retaining one or two additional N-terminal dipeptides (EA or EAEA), indicative of incomplete Ste13 processing (Fig. 6F, G). The most abundant species detected after deglycosylation corresponded to EA-*Mt*PhyA2, suggesting that a substantial fraction of the enzyme undergoes aberrant N-terminal processing during secretion in *K. phaffii*.

## Discussion

In this study, we identified three putative phytase sequences from the thermophilic fungus *M. thermophila* and expressed them heterologously in *K. phaffii* to evaluate their production and activity profiles. *Mt*PhyA2 and *Mt*PhyA3 exhibited high sequence similarity (98% identity) and comparable secretion levels and volumetric activities, whereas *Mt*PhyA1 showed lower sequence identity (25%) and predominantly accumulated intracellularly. Detailed biochemical characterization, including determination of pH and temperature optima, specific activity, and thermostability, was performed for *Mt*PhyA2, which exhibited the most favorable secretion profile and activity. All phytase constructs were initially integrated at the *HIS4* locus to ensure uniform production and secretion conditions. However, *his4*Δ strains are known to scale poorly under industrially relevant fermentation conditions [42, 43]. To overcome this limitation and improve production yields, the *P*_*CAT*_–MFα(Δ57–70)–*Mt*PhyA2 expression cassette was recloned into the pPpT4 vector for random genomic integration. Expression profiling (“landscaping”) of the resulting clones revealed a substantial increase in extracellular *Mt*PhyA2 secretion levels, and shake-flask evaluations of the top three strains demonstrated a 5–6-fold enhancement in phytase activity (U mL^− 1^ OD_600_^−1^) compared with the original *his4*Δ-integrated strain. Given the report by Navone et al. [44] who observed superior AppA expression from the methanol-inducible *AOX1* promoter compared with *P*_*CAT*_, we also tested *Mt*PhyA2 expression under *P*_*AOX1*_ control. Notably, the *AOX1* promoter performed markedly worse than the *CAT* promoter in our experimental setup, underscoring the importance of host–protein-specific promoter optimization. This may be due to limitations in the secretory pathway, where excessively high production levels can result in protein misfolding, intracellular retention, or secretion bottlenecks. Moderate expression driven by *P*_*CAT*_ may therefore enable more efficient processing and secretion.

Recombinant *Mt*PhyA3 (also referred to as rSt-Phy) was previously produced in *E. coli*, where it showed activity between pH 3.0 and 7.0 with an optimum at pH 5.0, and a temperature optimum of 60 °C [22]. In that study, the gene was directly amplified from the *M. thermophila* genome without codon optimization, and the enzyme accumulated insolubly, requiring refolding, most likely due to the inability of *E. coli* to form eukaryotic disulfide bonds, which often results in misfolded or unstable phytases. Along with other fungal phytases, *Mt*PhyA3 has also been heterologously produced in *Aspergillus niger* [45], demonstrating that members of this enzyme family can be efficiently secreted by fungal hosts. *Mt*PhyA2 and *Mt*PhyA3 were efficiently secreted in *K. phaffii* in our study, whereas *Mt*PhyA1 exhibited poor secretion. *Mt*PhyA2 was produced at high titers and could be analyzed comprehensively.

In our study, purified *Mt*PhyA2 demonstrated optimal activity at pH 4–7 and 55–65 °C. At 65 °C, *Mt*PhyA2 exhibited a specific activity of 210.5 U·mg⁻¹, which falls within the typical range reported for fungal phytases. Although this is lower than the specific activity reported for bacterial phytases such as AppA from *E. coli* (up to 3,165 U·mg⁻¹ [46], fungal phytases are generally characterized by advantageous properties including enhanced thermostability, broader pH tolerance, and beneficial glycosylation patterns that can improve stability during feed processing. Notably, the specific activity of *Mt*PhyA2 exceeds that of some commercial fungal phytases; for example, Natuphos^®^ - derived from *Aspergillus niger* var. *ficuum* - has a reported specific activity of approximately 100 U·mg⁻¹ with optimal activity at pH 2.5–5.0 and ~ 50 °C [45].

Thermostability was evaluated by measuring residual activity after short-term exposure to elevated temperatures. *Mt*PhyA2 retained substantial activity at 65 °C and 37 °C, with 60% and 70% of activity remaining, respectively, after heat treatment at up to 95 °C for 2 min. These results indicate pronounced thermal stability beyond its catalytic temperature optimum. Such stability is particularly relevant for industrial processes such as feed pelleting, where enzymes are exposed to transient high temperatures. The glycosylation pattern conferred by *K. phaffii* may further contribute to the enhanced thermal stability of *Mt*PhyA2. Together, these characteristics (broad pH and temperature tolerance, thermostability, high specific activity for a fungal enzyme, scalability, and efficient secretion by *K. phaffii*) highlight *Mt*PhyA2 as a promising candidate for industrial phytase production.

Following standard practice, we initially employed the *S. cerevisiae* MFα(Δ57–70) signal peptide to drive secretion of the *Mt*PhyA enzymes, as it is a well-established and robust leader sequence for heterologous protein export [47]. We used the truncated MFα(Δ57–70) variant described by [32], which typically outperforms the original Invitrogen version. Motivated by reports demonstrating the utility of Gcw C-terminal domains as display elements [48], we also evaluated pre-signal sequences from major Gcw proteins. Although pre-Gcw signal sequences enabled secretion of *Mt*PhyA2, overall yields reached only ~ 60–80% of those obtained with MFα(Δ57–70), and the slightly higher apparent molecular weight on SDS–PAGE suggests that these constructs may undergo incomplete or incorrect signal peptide processing. However, when fused to the MFα pro-region, the preGcw28–proMFα(Δ57–70) construct improved *Mt*PhyA2 secretion approximately twofold. Since Ost1-derived leaders have been shown to enhance secretion, particularly for proteins susceptible to vacuolar targeting [39], we additionally tested Ost1 signals from *K. phaffii*, *Ogataea polymorpha*, and *Kluyveromyces lactis*. Several of these, notably *Kp*Ost1–proMFα(Δ57–70), further increased secretion of *Mt*PhyA2. However, when applied to other recombinant proteins such as CalB and HRP, secretion performance was comparable to but did not succeed MFα(Δ57–70). This agrees with previous observations [50] highlighting that secretion efficiency is highly protein-specific and often not transferable across different targets.

Proteins secreted using the MFα signal sequence are typically processed in two steps: first, the Kex2 protease cleaves at the KR site, and subsequently, Ste13 removes the EAEA dipeptide repeats (reviewed in [49]). The incomplete processing of *Mt*PhyA2 observed in our study is consistent with previous reports showing that in *K. phaffii*, the endogenous Ste13 protease exhibits lower processing efficiency compared to its *S. cerevisiae* counterpart [50]. More strikingly, in cases where the EAEA motif was efficiently removed, we also observed the partial absence of the N-terminal glutamine (Q). This finding aligns with observations by Wyss et al. [45], who showed that N-terminal sequencing of *Mt*PhyA3 secreted by *Aspergillus niger* revealed a mature protein beginning with the sequence SESRP, rather than the predicted N-terminal Q based on the von Heijne rules (von Heijne 1983) or modern signal peptide prediction tools such as SignalP 6.0 [31, 51]. Our results therefore suggest that *K. phaffii* exhibits a similar processing pattern, not only removing the EAEA sequence via Ste13 but also cleaving off the subsequent glutamine residue. The protease responsible for this additional trimming step remains unidentified in both *A. niger* and *K. phaffii*. This conserved N-terminal processing event points to an as-yet uncharacterized proteolytic activity within the fungal secretory pathway.

Finally, bioreactor cultivation further increased *Mt*PhyA2 production, achieving secretion levels of 1.6 g L⁻¹ and demonstrating that *K. phaffii* enables efficient and readily scalable phytase manufacturing. Overall, *K. phaffii* has been widely used as a host for recombinant phytase production due to its ability to achieve high cell densities and efficient protein secretion. For example, expression of *Citrobacter amalonaticus* phytase in *K. phaffii* achieved volumetric activities of up to 35,032 U·mL⁻¹ and protein titers of 9.58 g·L⁻¹ in fed-batch fermentation following optimization of promoter strength, gene copy number, and secretion pathways [52]. The native *C. amalonaticus* phytase exhibits a specific activity of 3,548 U·mg⁻¹ [52], reflecting the generally higher catalytic efficiencies observed for bacterial phytases compared to fungal enzymes.

Since *Mt*PhyA2 titers were still increasing after 92 h under our initial bioreactor conditions, there is likely considerable potential for further improvement through reactor process optimization. Combined with its high catalytic efficiency and pronounced thermostability, matching or even surpassing the heat tolerance of the widely used *E. coli* AppA phytase, *Mt*PhyA2 offers promising properties for industrial applications. The use of a food-grade, GRAS-compatible production host such as *K. phaffii* further simplifies regulatory approval for feed and food applications, strengthening the commercial potential of *Mt*PhyA2. Beyond its relevance for monogastric animal nutrition, thermostable phytases like *Mt*PhyA2 may also be valuable in human dietary supplementation, organic phosphorus recovery from agricultural side streams, and pharmaceutical formulations requiring long-term enzyme stability. Their capacity to reduce undigested phytate excretion aligns with global efforts to lower phosphorus runoff and support environmentally responsible agriculture. Future developments could include engineering *K. phaffii* for even higher secretion efficiency, evaluating *Mt*PhyA2 performance in industrial feed matrices, and conducting techno-economic analyses to assess large-scale production feasibility. Together, these features highlight *Mt*PhyA2 as a promising next-generation fungal phytase for sustainable biotechnology.

## Data Availability

All original data are available from the authors of this publication upon reasonable request.
